# Identification of atrial fibrillation-related genes through transcriptome data analysis and Mendelian randomization

**DOI:** 10.3389/fcvm.2024.1414974

**Published:** 2024-07-11

**Authors:** Yujun Zhang, Qiufang Lian, Yanwu Nie, Wei Zhao

**Affiliations:** ^1^ Data Management Center, Xianyang Hospital, Yan'an University, Xianyang, China; ^2^ Department of Cardiology, Xianyang Hospital, Yan'an University, Xianyang, China; ^3^ School of Public Health, Nanchang University, Nanchang, China

**Keywords:** atrial fibrillation, transcriptomic data, Mendelian randomization, therapeutic targets, expression quantitative trait loci

## Abstract

**Background:**

Atrial fibrillation (AF) is a common persistent arrhythmia characterized by rapid and chaotic atrial electrical activity, potentially leading to severe complications such as thromboembolism, heart failure, and stroke, significantly affecting patient quality of life and safety. As the global population ages, the prevalence of AF is on the rise, placing considerable strains on individuals and healthcare systems. This study utilizes bioinformatics and Mendelian Randomization (MR) to analyze transcriptome data and genome-wide association study (GWAS) summary statistics, aiming to identify biomarkers causally associated with AF and explore their potential pathogenic pathways.

**Methods:**

We obtained AF microarray datasets GSE41177 and GSE79768 from the Gene Expression Omnibus (GEO) database, merged them, and corrected for batch effects to pinpoint differentially expressed genes (DEGs). We gathered exposure data from expression quantitative trait loci (eQTL) and outcome data from AF GWAS through the IEU Open GWAS database. We employed inverse variance weighting (IVW), MR-Egger, weighted median, and weighted model approaches for MR analysis to assess exposure-outcome causality. IVW was the primary method, supplemented by other techniques. The robustness of our results was evaluated using Cochran's Q test, MR-Egger intercept, MR-PRESSO, and leave-one-out sensitivity analysis. A “Veen” diagram visualized the overlap of DEGs with significant eQTL genes from MR analysis, referred to as common genes (CGs). Additional analyses, including Gene Ontology (GO) enrichment, Kyoto Encyclopedia of Genes and Genomes (KEGG) pathways, and immune cell infiltration studies, were conducted on these intersecting genes to reveal their roles in AF pathogenesis.

**Results:**

The combined dataset revealed 355 differentially expressed genes (DEGs), with 228 showing significant upregulation and 127 downregulated. Mendelian randomization (MR) analysis identified that the autocrine motility factor receptor (AMFR) [IVW: OR = 0.977; 95% CI, 0.956–0.998; *P* = 0.030], leucine aminopeptidase 3 (LAP3) [IVW: OR = 0.967; 95% CI, 0.934–0.997; *P* = 0.048], Rab acceptor 1 (RABAC1) [IVW: OR = 0.928; 95% CI, 0.875–0.985; *P* = 0.015], and tryptase beta 2 (TPSB2) [IVW: OR = 0.971; 95% CI, 0.943–0.999; *P* = 0.049] are associated with a reduced risk of atrial fibrillation (AF). Conversely, GTPase-activating SH3 domain-binding protein 2 (G3BP2) [IVW: OR = 1.030; 95% CI, 1.004–1.056; *P* = 0.024], integrin subunit beta 2 (ITGB2) [IVW: OR = 1.050; 95% CI, 1.017–1.084; *P* = 0.003], glutaminyl-peptide cyclotransferase (QPCT) [IVW: OR = 1.080; 95% CI, 1.010–0.997; *P* = 1.154], and tripartite motif containing 22 (TRIM22) [IVW: OR = 1.048; 95% CI, 1.003–1.095; *P* = 0.035] are positively associated with AF risk. Sensitivity analyses indicated a lack of heterogeneity or horizontal pleiotropy (*P* > 0.05), and leave-one-out analysis did not reveal any single nucleotide polymorphisms (SNPs) impacting the MR results significantly. GO and KEGG analyses showed that CG is involved in processes such as protein polyubiquitination, neutrophil degranulation, specific and tertiary granule formation, protein-macromolecule adaptor activity, molecular adaptor activity, and the SREBP signaling pathway, all significantly enriched. The analysis of immune cell infiltration demonstrated associations of CG with various immune cells, including plasma cells, CD8T cells, resting memory CD4T cells, regulatory T cells (Tregs), gamma delta T cells, activated NK cells, activated mast cells, and neutrophils.

**Conclusion:**

By integrating bioinformatics and MR approaches, genes such as AMFR, G3BP2, ITGB2, LAP3, QPCT, RABAC1, TPSB2, and TRIM22 are identified as causally linked to AF, enhancing our understanding of its molecular foundations. This strategy may facilitate the development of more precise biomarkers and therapeutic targets for AF diagnosis and treatment.

## 1 Introduction

Atrial fibrillation (AF), the most common persistent arrhythmia, is associated with heightened risks of stroke, heart failure, sudden death, and cardiogenic embolism, leading to increased hospitalizations and mortality rates (1). Presently, around 34 million people worldwide are affected by AF, a number projected to significantly increase owing to an aging global population and a rise in risk factors like obesity, hypertension, and diabetes mellitus. This escalation could boost the economic strain on individuals and societies by as much as 60% by the year 2050 (2). The sinoatrial node, the heart's primary pacemaker, is encased in a thin layer of epicardial adipose tissue. Its interior connects to the atrial myocardial layer, which primarily consists of pacemaker cells, transitional cells, and a few Purkinje fibers. It generates impulses that are transmitted to the atrial muscle and the atrioventricular node through the internodal pathways (3). Research has indicated that atrial fibrillation characterized by long RR intervals often results from a second-degree or higher atrioventricular conduction block. In patients experiencing atrial fibrillation and long RR intervals (Tachy-Brady syndrome), radiofrequency ablation can mitigate the effects of the vagus nerve, reduce concealed conduction in the atrioventricular node, restore sinoatrial node functionality, and successfully sustain sinus rhythm (4). This treatment eliminates the long intervals, obviating the need for pacemaker therapy. Numerous factors contribute to the development of atrial fibrillation (AF), such as cigarette smoking, alcohol consumption, hypertension, obesity, diabetes mellitus, heart attacks, heart failure, and other risk factors (5). Evidence from epidemiology shows that AF tends to run in families; having a parent with AF doubles the likelihood that their children will also develop this condition (6). The connections between specific genetic markers and the development of AF, however, remain poorly understood. Additionally, current drugs for treating AF often fall short in efficacy and carry potential for adverse effects (7). Compared to pharmacological approaches, ablation generally offers greater effectiveness but involves invasive techniques, possible complications, and a significant chance of recurrence over the long term (8). Thus, gaining a clearer understanding of AF's molecular mechanisms is essential for developing novel diagnostic biomarkers and therapeutic targets.

Recent progress in high-throughput gene chip and transcriptome sequencing technologies has enhanced their application in cardiovascular disease research. These technologies range from detecting copy number variations at the genomic level to exploring gene expression at the transcriptome level, with integrated bioinformatics emerging as a key approach for pinpointing potential biomarkers. For instance, Zhang et al. (9) identified CXCR4, IGFBP2, IGFBP3, and FHL2 as genes linked to the risk of AF using bioinformatics analysis. CXCR4, functioning as a receptor for CXCL12/SDF-1, significantly influences cellular growth, differentiation, stress responses, and inflammatory reactions. Studies have shown that myocardial cells in atrial fibrillation display elevated CXCR4 expression and heightened inflammatory responses compared to controls. This may promote inflammation via the PI3K/AKT signaling pathway, potentially leading to AF (10). IGFBP2, part of the insulin-like growth factor binding protein (IGFBP) family, is involved in cell migration, tumor invasion, cell proliferation, and tumor angiogenesis. Research indicates IGFBP2 inhibits PTEN and enhances PTEN tyrosine phosphorylation through dimerization with RPTPβ, thus fostering vascular smooth muscle cell growth (11). Given the role of the PTEN/AKT/mTOR pathway in cardiac hypertrophy and fibrosis (12), IGFBP2's impact on PTEN could affect AF development. It activates integrin β1 and downstream pathways, necessitating ILK for cell motility induction and NF-κB activation (13). NF-κB participates in the regulation of inflammatory cytokines, thrombosis, and fibrosis genes (14), suggesting the IGFBP2/integrin/ILK/NF-κB axis might play a role in the development and progression of AF. IGFBP3, the principal binding target for IGF-1, modulates cellular responses to IGF-1 either by forming specific complexes with or without IGF-1 signaling pathways, thus moderating free IGF-1 levels in the body and restricting IGF-1 signaling activation in target cells. Research by Liakouli et al. shows that IGFBP-3 may facilitate myocardial fibrosis by promoting mesenchymal cell proliferation and extracellular matrix deposition (15). Busch et al. found in the Study of Health in Pomerania (SHIP) that a low IGF-1/IGFBP-3 ratio correlates with a higher incidence of AF (16). FHL2 protein, as a functional partner of the α or β subunit of the delayed rectifier potassium current, regulates potassium ion channels and is essential for the delayed rectifier potassium current formation (17). Pape et al. have observed that expression of Kv1.5 (encoded by KCNA5), a major component of Ikur, is reduced in the atrial muscle of AF patients. Since Ikur is critical for the atrial muscle cell action potential repolarization phase (AERP) and highly selective to atrial tissue, its downregulation reduces APD frequency adaptability and the ERP/APD ratio, thus fostering the onset and persistence of atrial fibrillation (18, 19). In a similar vein, Wang et al. (20) discovered ZBTB20, ERBB4, CREB1, and BNIP3l as potential indicators for the early diagnosis and prospective treatment of AF. eQTLs, which are genetic variants that affect gene expression, play a pivotal role in elucidating biological pathways. They do so by linking genetic variations to gene expression changes and identifying pertinent biomarkers within GWAS (21). Recent GWAS on AF (22) have pinpointed around 140 genetic variant loci linked to the disorder. Yet, establishing a direct causal link between these markers and the pathophysiology of AF still requires more definitive evidence.

MR serves as an analytic tool to determine causal relationships between exposures and outcomes, using genetic variants as instrumental variables (IVs) (23). This method leverages the random distribution of alleles during meiosis, thus minimizing the influence of confounding factors that frequently affect traditional epidemiological studies (24). Additionally, the allocation of genotypes precedes disease onset, thereby precluding any possibility of reverse causation (25). In our research, we merged bioinformatics with MR to pinpoint differentially expressed eQTLs as potential biomarkers for diagnosing and treating AF. Our study further explored the mechanisms of AF pathogenesis through analyses of enrichment and immune cell infiltration.

## 2 Materials and methods

### 2.1 Study design

**Figure 1:**
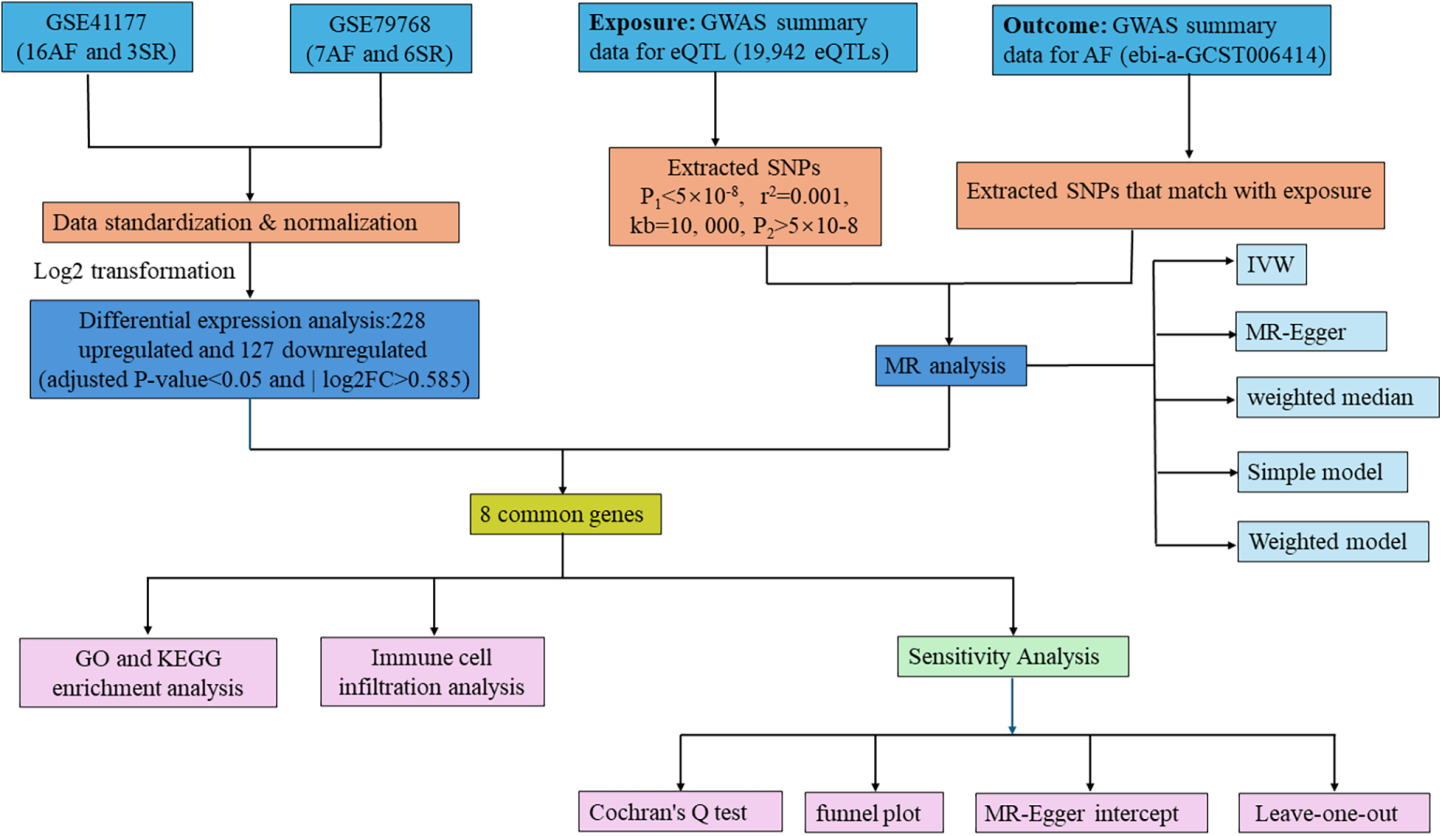
Flowchart of the study. AF, atrial fibrillation; SR, sinus rhythm; GWAS, genome-wide association study; GO, gene ontology; KEGG, Kyoto encyclopedia of genes and genomes; IVW, inverse variance weighted; MR, Mendelian randomization.

Initially, we extracted 355 DEGs from AF-related datasets GSE41177 and GSE79768. Following this, we applied two-sample MR using data from the GWAS (ebi-a-GCST006414) to explore the causal relationship between eQTL and AF. By overlapping DEGs and eQTLs showing significant results in MR analysis, we pinpointed CGs as prospective biomarkers for this study. MR studies must adhere to three fundamental criteria (26): (1) a strong association between the IV and the exposure, (2) no association of the IV with any confounders of the exposure-outcome relationship, and (3) the IV must impact the outcome solely through its influence on the exposure. We further ensured the integrity of our findings through sensitivity analyses. The study also included enrichment and immunohistochemistry analyses on the identified biomarkers, with the methodology summarized in Figure 1.

### 2.2 Data source

AF-related datasets were carefully selected from the GEO database based on specific criteria: (1) the datasets must comprise array data from human gene expression studies; (2) they should include samples from both AF patients and healthy controls; (3) they must come with sample information files; (4) both raw and processed data should be publicly accessible. Following these guidelines, we downloaded two microarray datasets, GSE41177 and GSE79768, both utilizing the Affymetrix Human Genome U133 Plus 2.0 Array [HG-U133_Plus_2] platform (27). GSE41177 consists of 23 cardiac tissue samples, including 16 from AF patients and 7 from individuals maintaining sinus rhythm (SR). GSE79768 includes 7 AF tissue samples and 6 SR samples. Additionally, we sourced GWAS summary statistics concerning eQTL and AF from the IEU Open GWAS project, collecting data on 19,942 eQTL datasets as exposures, with the specific AF-related dataset ebi-a-GCST006414 covering data from 977,323 individuals of European descent (47,309 cases and 930,014 controls). Since all data used in this study are publicly available and free to download, no separate ethical approval was necessary.

### 2.3 Data preprocessing and identification of DEGs

The process of data preprocessing and identifying differentially expressed genes (DEGs) began with the use of a Perl script to convert the probe matrix into a gene matrix, according to the annotation file. We excluded probes associated with multiple genes and calculated the average of multiple probes representing the same gene to establish the gene's final expression level (28). Next, we employed the “limma” package along with the combat function from the “sva” package to adjust for batch effects in the two datasets and to merge them (29, 30), revealing a total of 22,877 expressed genes. We then analyzed the DEGs between AF and SR tissues in the combined dataset using the “limma” package, selecting genes based on an adjusted *P*-value < 0.05 and an absolute log2 fold change (|log2FC|) greater than 0.585. Visualization of DEGs was achieved using the “pheatmap” and “ggplot2” R packages to produce heatmaps and volcano plots, respectively (31).

### 2.4 Selection of IVs

To enhance the accuracy and validity of our analysis on the causal link between eQTL and AF risk, we implemented rigorous quality control steps for selecting IVs. (1) SNPs significantly related to eQTL were chosen using a *P*-value threshold of <5 × 10^−8^ (24); (2) We set the linkage disequilibrium coefficient *R*^2 ^= 0.001and the linkage disequilibrium region width to 10,000 kb to maintain SNP independence (32); (3) SNPs directly associated with AF were excluded (*P* < 5 × 10^−8^) (33); (4) The *F*-statistic for each SNP was calculated using F= $\frac{R^{2}(n-2)}{1-R^{2}}$, where *n* is the sample size, and *R*^2^ is the proportion of variance in the exposure explained by SNPs, defined as *R*^2 ^= 2 × (1-MAF) × (MAF × 2), with MAF being the minor allele frequency and β the allele effect size, excluding SNPs with weak instrumental variables (*F* < 10) (34); (5) A harmonization process aligned effect directions and alleles, ensured SNPs had a minor allele frequency (>0.01), and removed palindromic and incompatible SNPs (35).

### 2.5 MR analysis

We evaluated the causal relationship between eQTL and AF using five distinct methods: IVW, weighted median (WM), MR-Egger, Simple model, and Weighted model. The IVW method, which aggregates Wald estimates from each SNP via meta-analysis, assumes no pleiotropy and is regarded as the most efficient method for causal estimation in this study (36). The MR-Egger method counters possible horizontal pleiotropy (indicated by an MR-Egger-intercept *P* < 0.05), providing a robust estimate (37). WM derives a median estimate from the distribution of individual SNP effect sizes, weighted by their precision, and offers reliable causal estimates especially when valid IVs constitute over 50% of the data (38). The Simple model withstands pleiotropic effects but may be less potent than IVW (39). The Weighted model adjusts for scenarios likely violating the pleiotropy assumption (40).

### 2.6 Sensitivity analysis

A series of sensitivity analyses were conducted to verify the robustness of our study findings: (1) Heterogeneity among IVs was assessed using Cochran's Q test for IVW and MR-Egger, with funnel plots visualizing significant heterogeneity *P* < 0.05 among the selected SNPs (41); (2) Horizontal pleiotropy within the MR study was evaluated through MR-Egger regression, where an MR-Egger intercept *P *< 0.05 indicated substantial horizontal pleiotropy (42) (3); The Leave-one-out approach was employed to sequentially exclude one SNP at a time and recalculate the MR estimates for the remaining SNPs to determine the impact of individual SNPs on the collective results (43, 44). These procedures were executed using the R software (version 4.1.2) with the “Two-Sample-MR” (version 0.5.6) and “MendelianRandomization” (version 0.4.3) R packages.

### 2.7 Enrichment analysis

Enrichment analysis for the CGs was performed using GO terms for molecular function (MF), biological process (BP), and cellular component (CC), as well as KEGG pathway analysis, utilizing the “clusterProfiler”, “org.Hs.eg.db”, and “enrichplot” R packages (45). Results were visually represented using the “ggplot2” R package, with a significance threshold of *P* < 0.05.

### 2.8 Prediction of miRNAs of intersecting genes

To explore the correlation between intersecting mRNAs and miRNAs, we used TargetScan, miRDB and miRanda databases to predict the interactions between miRNAs and intersecting genes, followed by Cytoscape analysis to construct a miRNA-mRNA networks.

### 2.9 Prediction of miRNAs of intersecting genes

To explore the correlation between intersecting mRNAs and miRNAs, we used TargetScan, miRDB and miRanda databases to predict the interactions between miRNAs and intersecting genes, followed by Cytoscape analysis to construct a miRNA-mRNA networks.

### 2.10 Immune cell infiltration analysis

Using the CIBERSORT algorithm, which applies an inverse convolution method to gene expression data, we estimated the abundance of 22 different immune cell types in cardiac tissues (46). *T*-tests compared the distribution of these cells between AF and SR samples. Correlations between the presence of characteristic genes and the infiltration of immune cells were analyzed using Spearman's correlation, and results were visualized using the “ggplot2” package, considering *P* < 0.05 as statistically significant.

## 3 Results

### 3.1 Identification of DEGs

**Figure 2:**
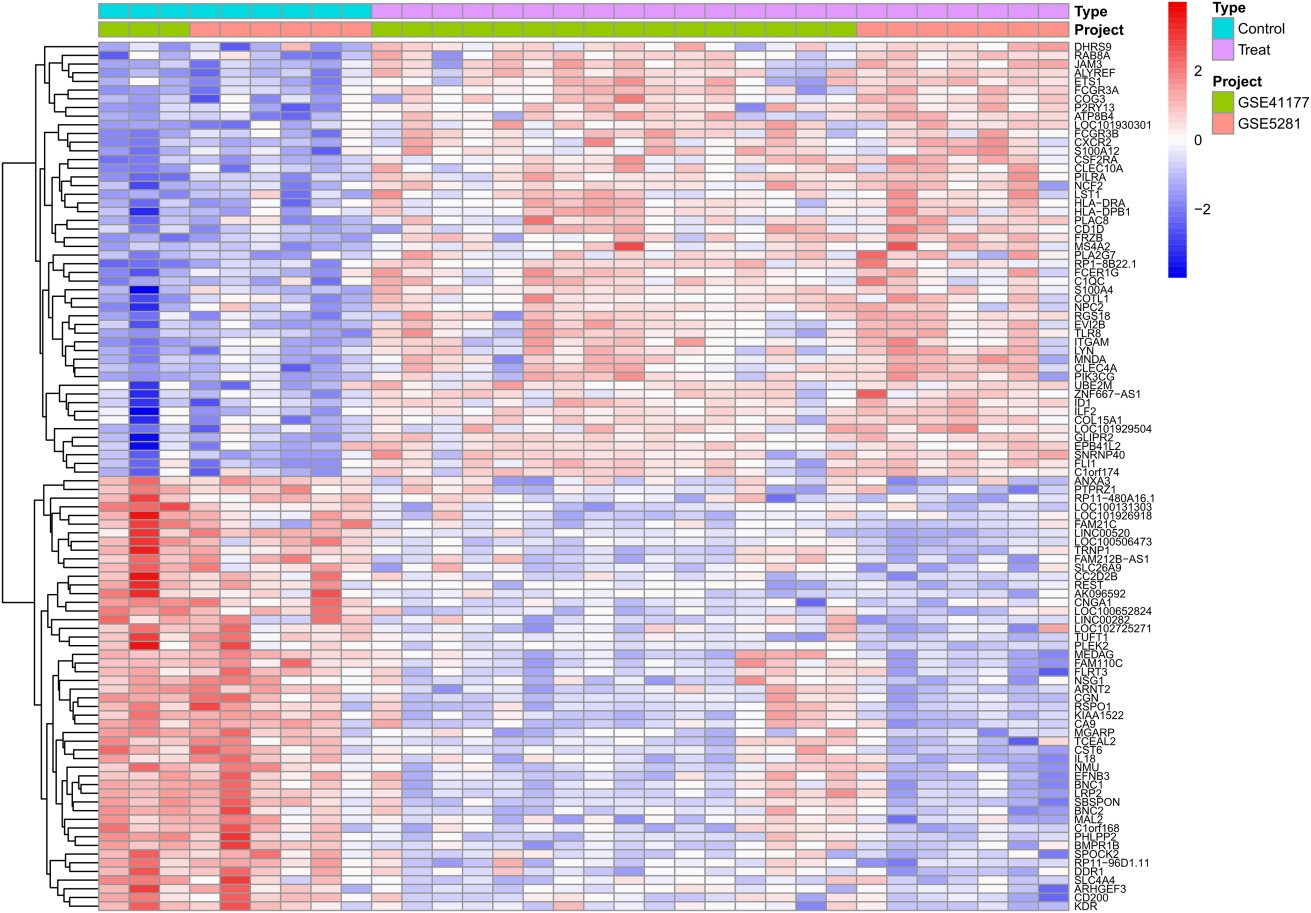
Differential expression gene heatmap. Red modules represent upregulated genes; blue modules represent downregulated genes.

We combined the expression profiles of AF and SR samples from datasets GSE41177 and GSE79768, resulting in a dataset comprising 22 AF and 9 SR samples. After normalizing to reduce batch effects, we identified 355 differentially expressed genes (DEGs) between the AF and SR tissues, with 228 genes up-regulated and 127 down-regulated, as detailed in Supplementary Table S1. The top 50 upregulated and downregulated DEGs are visually represented in a heatmap (Figure 2).

### 3.2 The causal effects of eQTL predicted by genetics on AF

**Figure 3:**
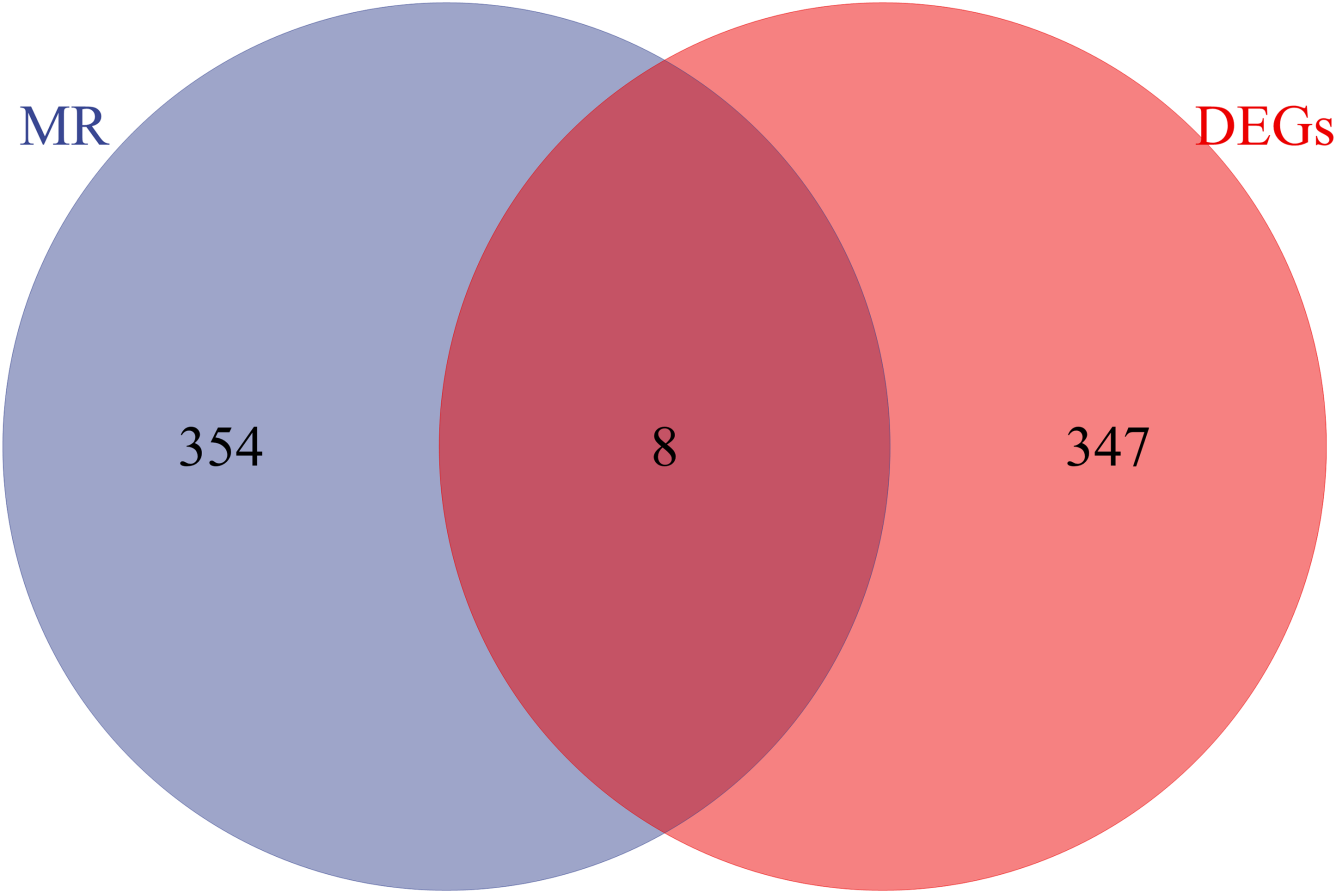
Venn diagram of the intersection of differentially expressed genes and eQTL genes in MR analysis.

**Figure 4:**
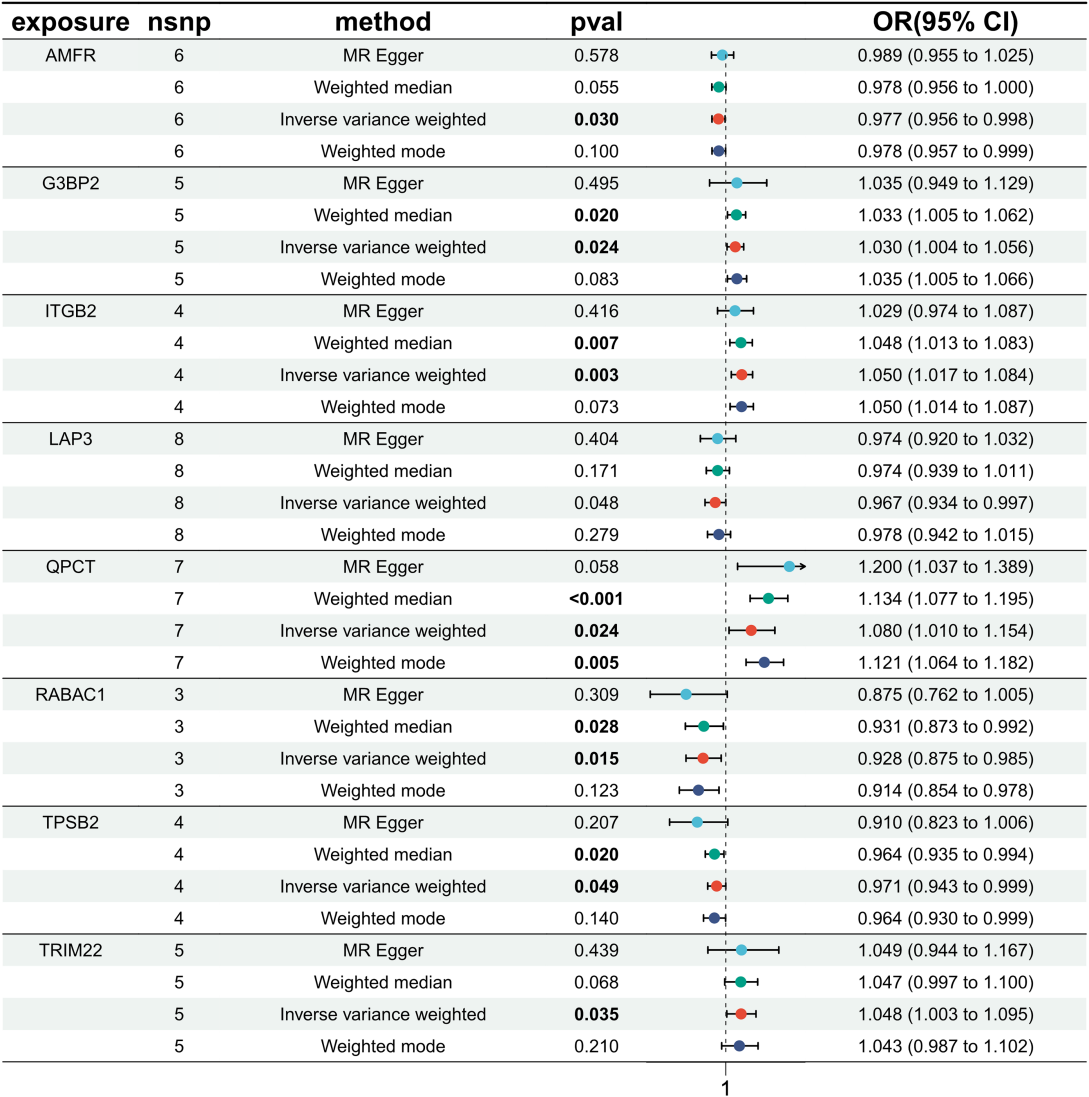
MR estimated the relationship between the 8 intersecting genes and AF. MR, Mendelian randomization; AF, atrial fibrillation; SNP, single nucleotide polymorphism; OR, odds ratios; CI, confidence interval.

Exploring the causal relationships between 19,942 eQTLs and AF, we discovered 1,811 eQTLs causally linked to the onset of AF. By overlaying these eQTLs with the 355 DEGs, we identified 8 candidate genes (CGs) shown in Figure 3: automatic mobility factor receptor (AMFR), leucine aminopeptidase 3 (LAP3), Rab acceptor 1 (RABAC1), tryptase beta 2 (TPSB2), GTPase activating SH3 domain binding protein 2 (G3BP2), integrin subunit beta 2 (ITGB2), Glutaminyl Pettide Cycle transfer (QPCT), and (TRIM22). IVW analysis indicated that AMFR [OR = 0.977; 95% CI, 0.956–0.998; *P *= 0.030], LAP3 [OR = 0.967; 95% CI, 0.934 0.997; *P *= 0.048], RABAC1 [OR = 0.928; 95% CI, 0.875 0.985; *P *= 0.015], and TPSB2 [IVW: OR = 0.971; 95% CI, 0.943 0.999; *P *= 0.049] were inversely correlated with AF risk. Conversely, G3BP2 [OR = 1.030; 95% CI, 1.004–1.056; *P *= 0.024], ITGB2 [OR = 1.050; 95% CI, 1.017–1.084; *P *= 0.003], QPCT [OR = 1.080; 95% CI, 1.010–0.997; *P *= 1.154], and TRIM22 [IVW: OR = 1.048; 95% CI, 1.003–1.095; *P *= 0.035] were associated with an increased risk of AF. The WM, MR-Egger, Simple model, and Weighted model analyses corroborated the direction of the IVW results (Figure 4).

### 3.3 Sensitivity analysis

Sensitivity analysis revealed no evidence of potential heterogeneity or horizontal pleiotropy within this study (Table 1). Cochran's Q-test was employed to assess the heterogeneity of estimated instrumental variables arising from individual genetic variations. The findings indicated that only QPCT demonstrated significant heterogeneity (*P *< 0.05), while other variables did not. Nevertheless, the application of the random-effects IVW model mitigated the impact of heterogeneity on the study's outcomes. The symmetry observed in the funnel plots’ dispersion of causal associations suggests the absence of confounding factors affecting the causal effect of the eQTL expression level on AF (Supplementary Figure S1). Furthermore, the MR-Egger intercept test, with a *P*-value >0.05, confirmed the absence of horizontal pleiotropy (Supplementary Figure S2). The “leave-one-out” analysis demonstrated that the exclusion of any single SNP did not significantly alter the MR Analysis results, affirming the robustness of our findings (Supplementary Figure S3).

**Table 1:** Heterogeneity and pleiotropy test between intersecting genes and atrial fibrillation.

Exposures	Heterogeneity test						Pleiotropy test		
	MR-Egger			Inverse variance-weighted			MR-Egger		
	Q	Q_df	Q_P	Q	Q_df	Q_P	Intercept	SE	P
AMFR	2.359	4	0.670	3.12	5	0.682	−0.006	0.007	0.432
LAP3	8.992	6	0.174	9.166	7	0.241	0.002	0.007	0.745
RABAC1	0.132	1	0.717	1.000	2	0.607	0.015	0.017	0.523
TPSB2	0.244	2	0.885	1.994	3	0.574	0.023	0.018	0.317
G3BP2	3.657	3	0.301	3.675	4	0.452	−0.002	0.018	0.913
ITGB2	0.585	2	0.747	1.391	3	0.708	0.007	0.008	0.464
QPCT	10.058	5	0.074	14.916	6	0.021	−0.016	0.010	0.181
TRIM22	3.470	3	0.325	3.470	4	0.482	−0.0002	0.009	0.982

df, degree of freedom; MR, Mendelian randomization; Q, heterogeneity statistic Q.

### 3.4 GO and KEGG enrichment analysis of CGs

**Figure 5:**
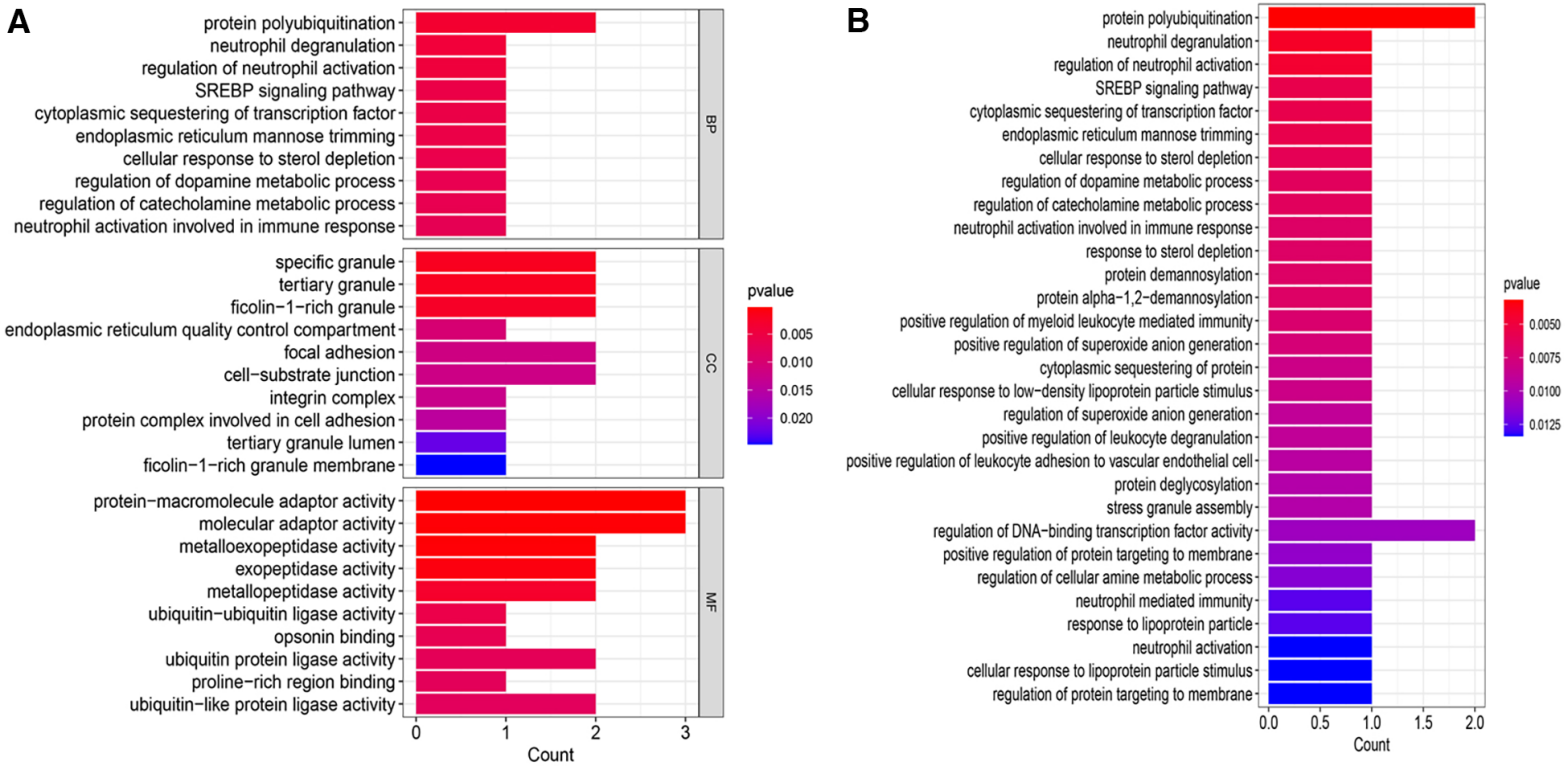
Enrichment analysis. (**A**) GO enrichment analysis bar chart. (**B**) KEGG enrichment analysis bar chart. BP, biological process; CC, cellular component; MF, molecular function.

GO and KEGG enrichment analyses were conducted to uncover the biological processes and pathways associated with the 8 CGs (Figure 5). In terms of biological processes, the genes are primarily involved in protein polycysteinization, neutrophil degranulation, and neutrophil activation linked to immune responses. Cellular component analysis localized the genes to specific granules and ficolin-1-rich granules, among others. Molecular function focused on activities like metalloexopeptidase and exopeptidase activity. KEGG pathway analysis highlighted significant involvement in the SREBP signaling pathway.

### 3.5 miRNA-mRNA network construction

**Figure 6:**
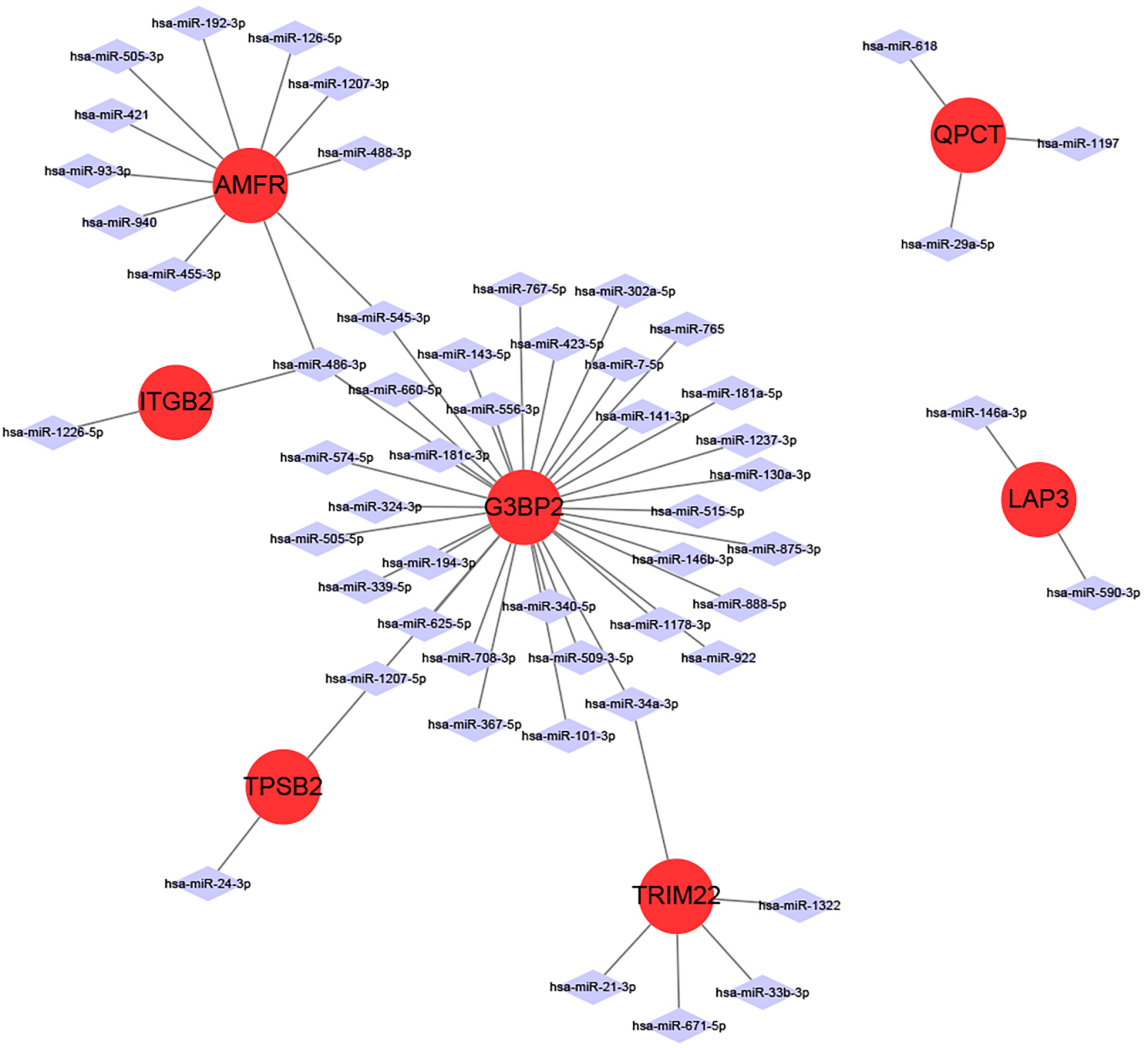
miRNA-mRNA network diagram.

miRNAs are a class of small non-coding RNA molecules that can regulate gene expression. In our study, we analyzed the interactions between eight intersecting genes and miRNAs using TargetScan, miRDB and miRanda databases databases, respectively, in order to construct mRNA-miRNA networks (Figure 6). We identified 7 intersecting genes (AMFR, LAP3, TPSB2, G3BP2, ITGB2, QPCT, and TRIM22) that could regulate miRNAs. In addition, we obtained a total of 55 miRNAs regulating intersecting genes, 11 regulating AMFR, 2 regulating LAP3, 2 regulating TPSB2, 34 regulating G3BP2, 2 regulating ITGB2, 3 regulating QPCT, and 5 regulating TRIM22.

### 3.6 Analysis of immune cell infiltration

**Figure 7:**
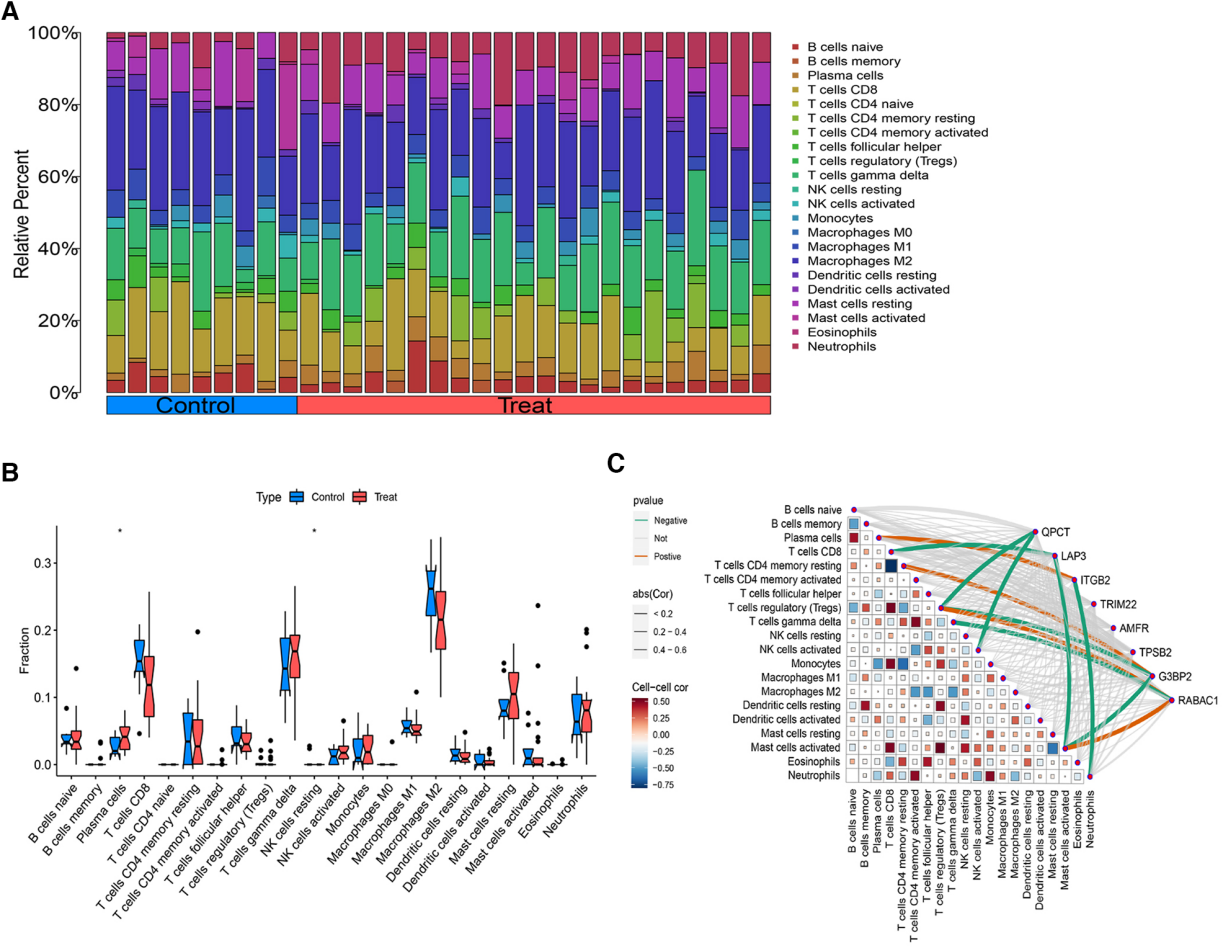
Analysis of immune cell infiltration. (**A**) The distribution of immune cells in each sample. (**B**) Differentially expressed immune cells between AF and SR samples. (**C**) Correlation analysis between immune cells and the 8 intersecting genes.

Using the CIBERSORT algorithm, we quantified the immune cell infiltration in AF and SR diagnosed patients, illustrated in Figure 7A. The activity of 22 immune cell types was analyzed between both groups, revealing that plasma cells and NK cells exhibited differential expression in AF and SR patients (*P *< 0.05), with both showing higher expression levels in the AF cohort (Figure 7B). Spearman correlation analysis investigated the relationships between subsets of immune cells and 8 CGs (Figure 7C). The analysis identified that QPCT expression was inversely associated with Tregs (*r* = −0.463, *P *= 0.042) and activated NK cells (*r* = −0.501, *P *= 0.018). LAP3 expression levels were negatively correlated with CD8+ T cells (*r* = −0.500, *P* = 0.018) and activated Mast cells (*r* = −0.505, *P *= 0.017). ITGB2 showed a positive correlation with Plasma cells (*r* = 0.467, *P *= 0.030) and an inverse relationship with Neutrophils (*r* = −0.452, *P *= 0.036). G3BP2 was positively associated with CD4+ memory resting T cells (*r* = 0.464, *P *= 0.029), but negatively with activated Mast cells (*r* = −0.443, *P *= 0.039). RABAC1 displayed a positive correlation with Tregs (*r* = 0.440, *P *= 0.040) and activated Mast cells (*r* = 0.596, *P *= 0.003), whereas it was negatively associated with gamma delta T cells (*r* = −0.445, *P *= 0.038). TRIM22, AMFR, and RPSB2 showed no significant correlations with any immune cell types.

## 4 Discussion

AF is a prevalent cardiovascular condition with an incidence that escalates with age. Recent advancements have led to the identification of numerous novel biomarkers for the early diagnosis, mechanistic exploration, and drug target identification in AF. Nonetheless, our comprehension of AF's genetic underpinnings remains markedly insufficient. Consequently, there is a critical necessity to unravel AF's pathogenesis and identify viable diagnostic and therapeutic targets to formulate an effective framework for its prevention, early detection, and management. In this research, we employed a holistic approach, integrating bioinformatics analysis with GWAS data mining, to identify 8 genes: AMFR, LAP3, RABAC1, TPSB2, G3BP2, ITGB2, QPCT, and TRIM22—implicated in AF's etiology. Through functional enrichment analysis, we discovered these genes predominantly engage in protein polyubiquitination and neutrophil degranulation, with notable enrichment in pathways such as the SREBP signaling pathway. Furthermore, our investigation into immune cell infiltration revealed that plasma cells and NK cells were abundantly expressed in AF patients. Notably, among the 8 genes, QPCT, LAP3, ITGB2, G3BP2, and RABAC1 exhibited correlations with diverse immune cells.

The AMFR, also known as gp78, is a receptor that binds to AMF ligands, facilitating signal transduction through the activation of G protein-coupled receptors and tyrosine kinases. It plays a pivotal role in the recognition of misfolded proteins, marking them for ubiquitination and subsequent degradation by the proteasome through the endoplasmic reticulum-associated degradation (ERAD) pathway. This process prevents the accumulation of misfolded proteins within the endoplasmic reticulum lumen, thereby maintaining the integrity of the secretory pathway, an essential protective mechanism *in vivo* (47). In this study, we present the first evidence that AMFR may lower the incidence of AF and explore the expression profile of AMFR to elucidate its mechanism of action in AF further. Leucine aminopeptidases (LAPs), a category of zinc-dependent metalloenzymes, play a crucial role in protein metabolism. LAP3, a significant LAP member, specializes in the cleavage of N-terminal amino acid polypeptides, facilitating peptide bond hydrolysis (48). Its function involves cleaving proteins into smaller peptide molecules via the ubiquitin-proteasome pathway, which is critical in the antigen-presenting intracellular terminal phase of protein degradation. To our knowledge, the regulatory role of LAP3 in AF development remains unreported. RABAC1 encodes the membrane protein PRA1, which is instrumental in protein transport and has been shown to interact with various Rab GTPases (49). Rab proteins are known to bind tightly to GDP Dissociation inhibitor (GDI), forming complexes. This observation has led to the hypothesis that an effector, known as the GDI displacement factor (GDF), exists at the donor membrane. This factor is believed to play a crucial role in the dissociation of Rab-isoprenoid base tails and their reinsertion into the donor membrane (50). In vivo studies suggests that PRA1 knockout results in abnormal ER and Golgi apparatus phenotypes, indicating a more structural role in the early secretory pathway (51). However, the precise function of PRA1 in AF remains to be elucidated. In this study, by screening genes causally associated with AF through eQTL data and intersecting them with AF-associated DEGs from bioinformatics analysis, we confirmed that RABAC1 is overexpressed in AF patients and may serve as a protective factor in reducing morbidity. G3BP2, belonging to the RNA-binding protein family, is a critical component of stress granule assembly. It regulates gene expression in response to various environmental stimuli, affecting cell proliferation, protein degradation, and RNA stability (52). G3BP family proteins play roles in various diseases, including cancer, viral infections, Alzheimer's disease, and cardiovascular diseases, exhibiting diverse biological functions such as RNA metabolism, stress granule formation, signaling, cell cycle regulation, and protein ubiquitination degradation (53), however, the precise mechanisms underlying these processes require further exploration. Recent studies have indicated that G3BPs exhibit abnormal expression levels in cardiovascular diseases, with their mechanism of action primarily linked to stress granule formation. Recent studies have demonstrated that the protein encoded by the G3BP2 gene interacts with the NF-κB pathway (54). NF-κB, a crucial regulator of inflammatory responses, remains inactive when bound to IκB proteins. Upon stimulation, it dissociates from IκB and translocates to the nucleus to regulate the expression of target genes for inflammatory cytokines such as TNF-α, IL-1β, and IL-6, thereby initiating an inflammatory cascade. Ye et al. reported that inhibiting the NF-κB pathway with 5,7-dihydroxyflavone could reduce levels of pro-inflammatory cytokines, potentially decreasing susceptibility to AF (55). Additional research has shown that colchicine can lower rat susceptibility to AF by reducing inflammation-mediated atrial fibrosis (56). NF-κB is pivotal in gene transcription, noted for its rapid transcriptional activation (57). Previous studies have also demonstrated that AF in diabetic rats induced by thiazolidinediones was associated with activation of the NF-κB pathway, leading to a pro-inflammatory state, myocardial hypertrophy, and fibrosis (58). Therefore, it is hypothesized that G3BP2 may promote the onset of AF by interacting with the NF-κB pathway and triggering an inflammatory response. ITGB2, a complex heterodimer composed of α and β chains, functions as an integral cell membrane receptor, facilitating a multifaceted role in extracellular matrix (ECM)-cytoskeletal linkage and biochemical and mechanical signal transduction between the cell and its surroundings (59). Friedrichs et al. (60) demonstrated that ITGB2-mediated infiltration of polymorphonuclear neutrophils contributes to atrial fibrosis, thereby heightening susceptibility to AF in angiotensin II treated mice. Previous research has illustrated that ITGB2 enhances the cross-cell migration of white blood cells in both animal and cell culture experiments, impairing endothelial barrier integrity and participating in atherosclerosis development (61). As a risk factor, ITGB2 has been implicated in accelerating myocardial infarction and arterial thrombotic cerebral infarction via cell adhesion molecular pathways (62). This analysis further posits that ITGB2 could act as a risk factor, increasing AF incidence. The QPCT gene encodes glutaminyl cyclase, an enzyme responsible for the post-translationally modification of proteins by converting N-terminal glutamate into pyroglutamate. This modification increases protein resistance to protease degradation, enhances hydrophobicity and neurotoxicity, and predisposes the proteins to aggregation (63). Yamada et al. (64) demonstrated a correlation between the expression of the QPCT gene and hypertension in Japanese men. To date, research on QPCT in AF has been sparse, with its underlying molecular mechanisms yet to be fully elucidated. The TRIM protein family, prevalent across multicellular animals, functions as immunomodulatory proteins and E3 ubiquitin ligases (65). TRIM22, a member of this family, plays a critical role in modulating the body's immune-inflammatory response. Several studies have highlighted the significance of the inflammatory mechanism in the pathogenesis and progression of AF (66). Clinical and experimental research has identified elevated levels of serum inflammatory biomarkers and cardiac tissue inflammatory markers or cytokines/growth factors in individuals with AF (67). Inflammatory responses in the atrial wall can lead to increased oxidative stress, cardiomyocyte apoptosis, fibrosis, and abnormalities in gap junction regulation and intracellular calcium handling, potentially contributing to the development of arrhythmias and AF (68). TRIM22 has been implicated in the development of various cancers, including gastric (69), ovarian (70), and cervical cancer (71). Numerous epidemiological studies have established a strong link between malignant tumors and atrial fibrillation (AF), noting a higher prevalence of AF among cancer patients compared to those without cancer. A case-control study reported an AF incidence of 7.4% among patients diagnosed with tumors, which is higher than the 6.8% observed in the non-tumor group (72). Additionally, a long-term prospective study lasting 16.3 years showed an increased risk of AF in cancer patients, with a hazard ratio (HR) of 2.47 (73). Similarly, a large meta-analysis involving 5,889,234 patients found a 47% increased risk of AF in patients with malignant tumors within 90 days of diagnosis, compared to those without malignant tumors (74). Therefore, early prevention, recognition, and management of AF should be prioritized in populations with malignant tumors.

Our further exploration into the biological functions and key pathways of the 8 CGs revealed significant insights through GO enrichment analysis. These genes are predominantly associated with protein polyubiquitination and neutrophil-related pathways, including neutrophil degranulation, regulation of neutrophil activation, and neutrophil activation within the immune response, all of which were notably enriched. Protein polyubiquitination, a prevalent post-translational modification, involves a complex enzyme-linked reaction sequence utilizing ubiquitin-activating enzyme E1, ubiquitin-binding enzyme E2, and ubiquitin ligase E3 (75). Several E3 ubiquitin ligases, such as WWP2, SMURF1, and SMURF2, have been identified as key regulators in myocardial fibrosis (76). Steinle et al. (77) observed that both protein modification and neutrophil activation play roles in systemic inflammation and oxidative stress. The KEGG pathway analysis highlighte the SREBP signaling pathway, cytoplasmic sequestering of transcription factors, endoplasmic reticulum mannose trimming, and the cellular response to sterol depletion as main components.

We have also determined the effects of mRNA and miRNA gene interactions on disease. miRNAs have been shown to modulate a wide range of signaling pathways and cellular processes and to participate in intercellular communication for their biological functions (78). Recent studies have shown that mi RNAs control the function of various cells in the heart, such as cardiac myocytes, endothelial cells, smooth muscle cells, and fibroblasts, and play an important role in cardiovascular diseases, such as myocardial infarction, myocardial hypertrophy, fibrosis, cardiac failure, cardiac arrhythmia, inflammation, and atherosclerosis (79). Some miRNAs, including miRNA-21, miRNA-26, miRNA-29, and miRNA-133, have been reported to contribute to the process of atrial fibrosis by participating in the regulation of different signaling pathways (80), which suggests that miRNAs may play an important role in promoting the maintenance of AF.

Inflammation has been identified as a key risk factor for AF, with the immune response it triggers playing a crucial role in the initiation and progression of AF. Litviňuková et al. (81) discovered a substantial presence of immune cells in the atrial tissues of 14 donated hearts, with immune cells constituting 10.4% of the atrial samples. This finding underscores the significance of local immune cells in cardiac pathophysiological alterations. Wu et al. (82) found that CD8+ T cells were significantly more prevalent in AF patients than in individuals with normal rhythm. Zhou et al. (83) observed that macrophage M2, activated NK cells, and neutrophils might be implicated in the pathogenesis of AF. Our findings indicate an increased expression of plasma cells and NK cells in AF patients. Plasma cells, or effector B cells, are involved in synthesizing and storing antibodies and play a part in humoral immune responses, appearing in various diseases such as lung adenocarcinoma, myeloma, and SLE (84–86). NK cells, located in lymphoid organs and peripheral tissues, can influence disease progression at inflammatory sites through cytokine and chemokine production, cell-to-cell interactions, and engagements with other immune cells. However, the precise mechanisms through which inflammation impacts AF remain elusive. Current research suggests that inflammation contributes to atrial remodeling via oxidative stress. Structural and electrophysiological remodeling of the atrium is widely regarded as pivotal in the pathophysiology of AF, thereby influencing the condition's persistence and recurrence.

We investigated the interactions between AMFR, LAP3, RABAC1, TPSB2, G3BP2, ITGB2, QPCT, TRIM22, and immune cell infiltration in greater depth. QPCT was found to inversely correlate with Tregs and activated NK cells. Similarly, LAP3 was inversely related to CD8+ T cells and activated Mast cells, while ITGB2 displayed a positive relationship with Plasma cells and a negative one with Neutrophils. G3BP2 correlated positively with CD4+ memory resting T cells and negatively with activated Mast cells. RABAC1 exhibited positive correlations with Tregs and activated Mast cells, yet it had an inverse relationship with gamma delta T cells. In contrast, TRIM22, AMFR, and TPSB2 showed no discernible correlations with immune cells. These results highlight the complex interactions between AF-related genes and immune cells, emphasizing the importance of further exploration into these dynamics. Notably, QPCT, LAP3, ITGB2, G3BP2, and RABAC1 are identified as crucial in AF pathogenesis through their modulation of immune infiltration.

This study offers several advantages. Primarily, this study is the first, to our knowledge, to integrate bioinformatics with MR for the analysis of AF biomarkers, revealing a causal link between 8 CGs and AF. Furthermore, the utilization of MR Analysis ensures that our results are robust against reverse causality. Additionally, we conducted multiple sensitivity analyses to verify the consistency of the causal estimates, affirming the reliability of our conclusions. However, there are limitations to our study: (1) The microarray analysis involved a small sample size, which could introduce bias and underscores the necessity for more extensive future studies. (2) Although we merged two GEO datasets and identified eight CGs by intersecting genes significant in both DEGs and MR Analysis, the exact roles of these CGs in AF require further elucidation through *in vitro* and *in vivo* studies. (3) Since our sample solely comprised individuals from a European background, this may limit the generalizability of our findings to other ethnic groups.

In summary, our study leveraged bioinformatics and MR Analysis to investigate genes associated with AF, identifying 8 genes with causal links to the onset and progression of AF. AMFR, LAP3, RABAC1, and TPSB2 were identified as protective factors that could potentially reduce the incidence of AF, while G3BP2, ITGB2, QPCT, and TRIM22 were deemed risk factors that could promote the disease's development. Furthermore, GO and KEGG enrichment analyses revealed that these 8 GCs were predominantly involved in protein ubiquitination and the SREBP signaling pathway. Analysis of the association between gene expression and immune cell infiltration highlighted that LAP3, RABAC1, G3BP2, ITGB2, and QPCT might influence AF through the regulation of immune cell infiltration. These findings suggest that the genes identified could serve as novel biomarkers and potential therapeutic targets for AF, offering insights into the molecular mechanisms underlying AF pathology.

## Data Availability

The datasets presented in this study can be found in online repositories. The names of the repository/repositories and accession number(s) can be found in the article/**Supplementary Material**.
