# Supplementary material for: Identification of atrial fibrillation-related genes through transcriptome data analysis and Mendelian randomization
Source: Front Cardiovasc Med. 2024 Jul 11;11:1414974. doi: 10.3389/fcvm.2024.1414974 (PMC11269132; doi:10.3389/fcvm.2024.1414974)
Supplement: Supplementary file 2 [file Datasheet1.pdf]

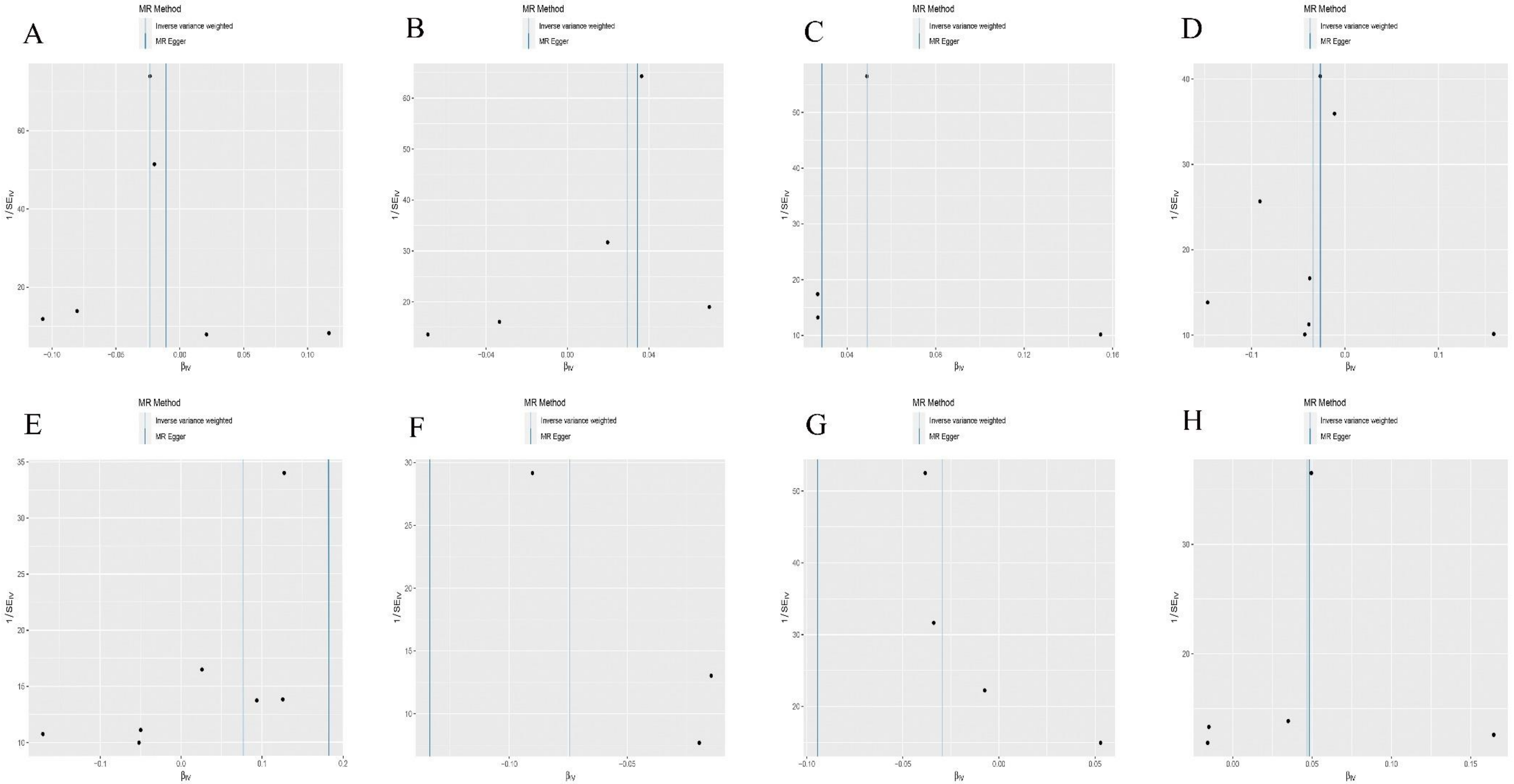

Figure S1 Funnel plots to the causal association of 8 CGs on Atrial fibrillation. (A) Funnel plot of the causal effect of AMFR on coronary artery disease. (B) Funnel plot of the causal effect of G3BP2 on Atrial fibrillation. (C)Funnel plot of the causal effect of ITGB2 on Atrial fibrillation.

(D)Funnel plot of the causal effect of LAP3 on Atrial fibrillation. (E) Funnel plot of the causal effect of QPCT on Atrial fibrillation. (F) Funnel plot of the causal effect of RABAC1 on Atrial fibrillation. (G) Funnel plot of the causal effect of TPSB2 on co Atrial fibrillation. (H) Funnel plot of the causal effect of TRIM22 co Atrial fibrillation.
