# Supplementary material for: Identification of atrial fibrillation-related genes through transcriptome data analysis and Mendelian randomization
Source: Front Cardiovasc Med. 2024 Jul 11;11:1414974. doi: 10.3389/fcvm.2024.1414974 (PMC11269132; doi:10.3389/fcvm.2024.1414974)
Supplement: Supplementary file 3 [file Datasheet2.pdf]

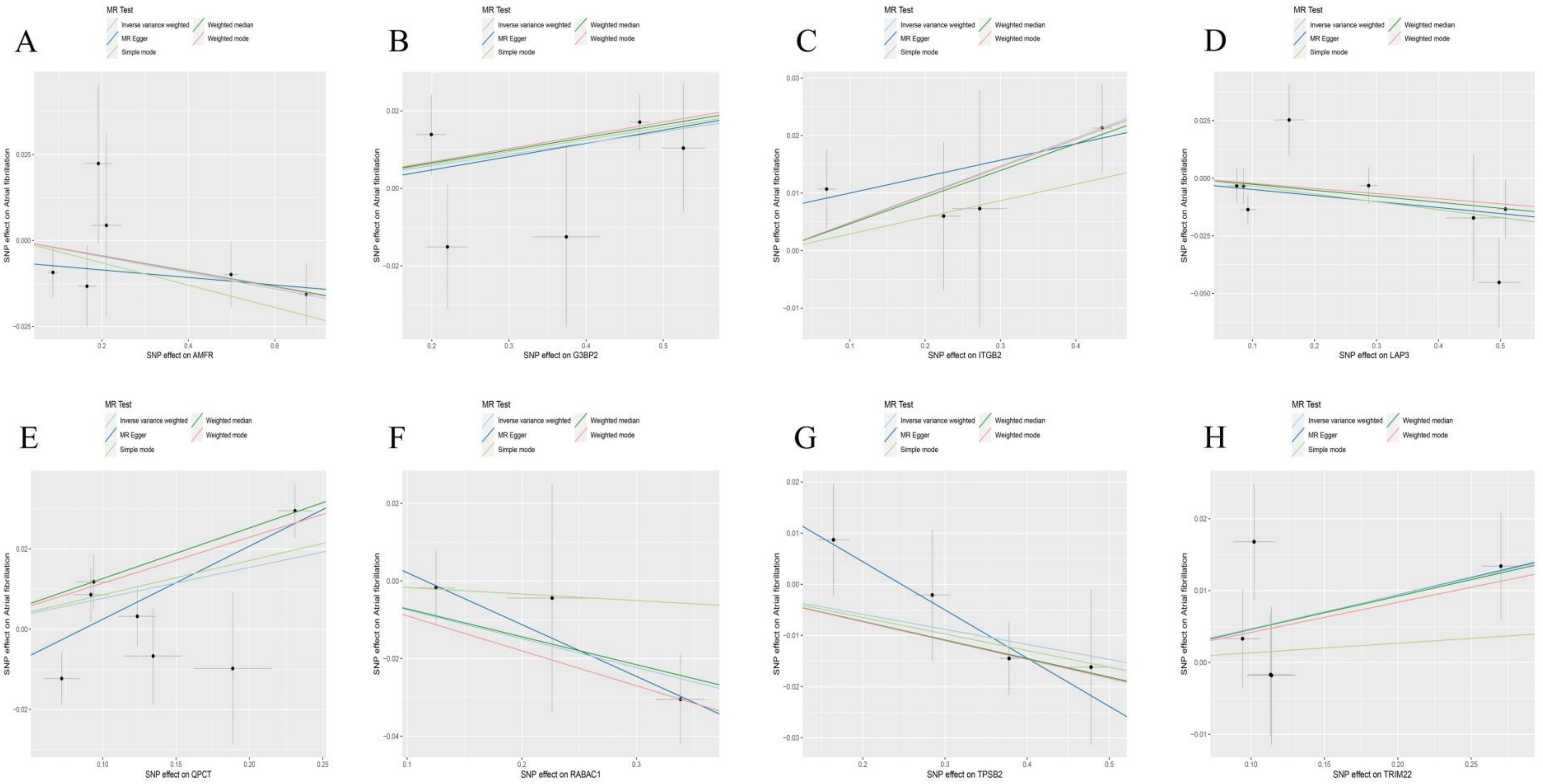

Figure S2 Scatter plots of 8CGs on atrial fibrillation. (A) Scatter plots of AMFR atrial fibrillation. (B) Scatter plots of G3BP2 on atrial fibrillation. (C) Scatter plots of ITGB2 on atrial fibrillation. (D)Scatter plots of LAP3 on atrial fibrillation. (E) Scatter plots of QPCT on atrial fibrillation. (F) Scatter plots of RABAC1 on atrial fibrillation. (G) Scatter plots of TPSB2 on atrial fibrillation. (H) Scatter plots of TRIM22 on

atrial fibrillation.
