# Supplementary material for: Identification of atrial fibrillation-related genes through transcriptome data analysis and Mendelian randomization
Source: Front Cardiovasc Med. 2024 Jul 11;11:1414974. doi: 10.3389/fcvm.2024.1414974 (PMC11269132; doi:10.3389/fcvm.2024.1414974)
Supplement: Supplementary file 4 [file Datasheet3.pdf]

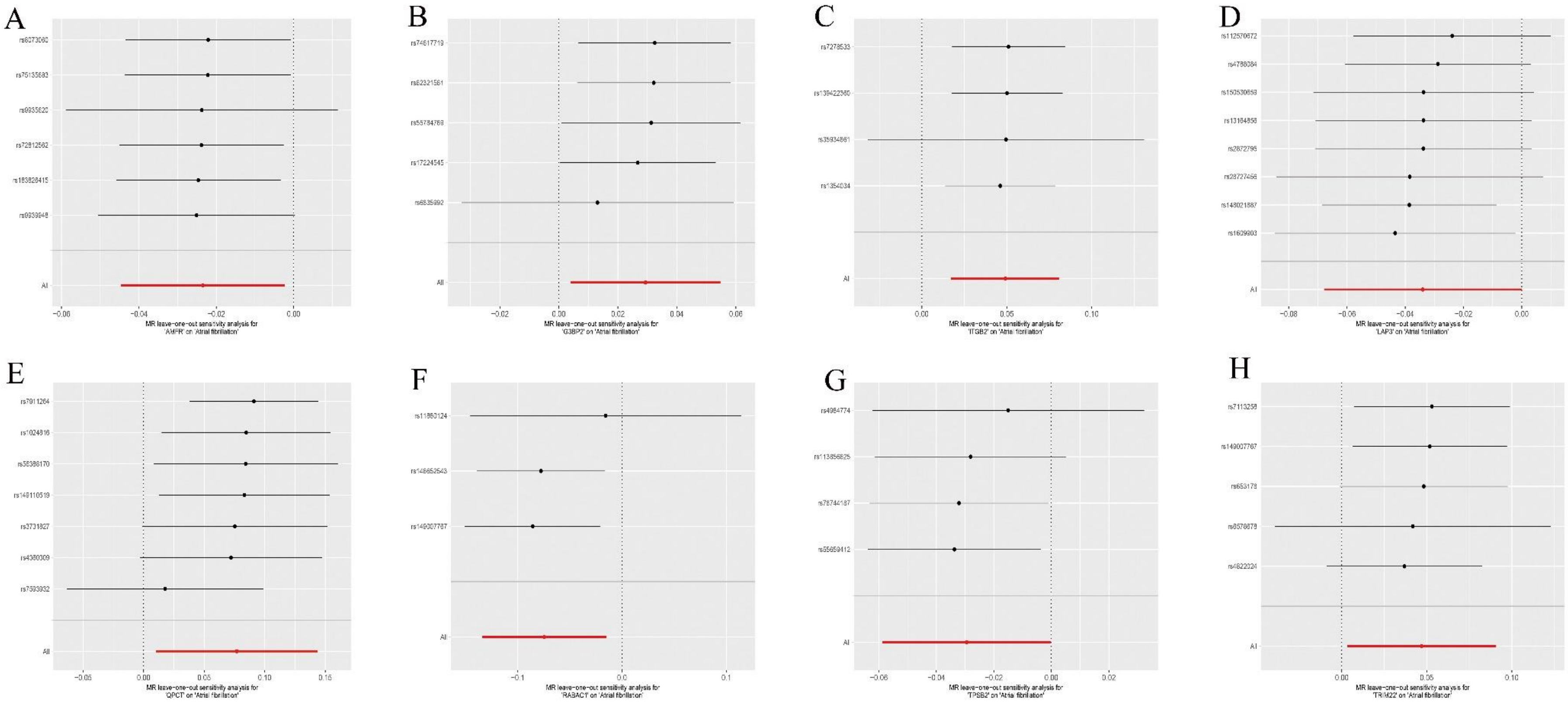


**Figure S3** Forest map of the analysis results using the "leave-one-out". (A) Leave-one-out analysis plots for AMFR on the risk of atrial fibrillation. (B) Leave-one-out analysis plots for G3BP2 on the risk of atrial fibrillation. (C)Leave-one-out analysis plots for ITGB2 on the risk of atrial fibrillation. (D) Leave-one-out analysis plots for LAP3 on the risk of atrial fibrillation. (E) Leave-one-out analysis plots for QPCT on the risk of atrial fibrillation. (F) Leave-one-out analysis plots for RABAC1 on the risk of atrial fibrillation. (G) Leave-one-out analysis plots for TPSB2 on the risk of atrial fibrillation. (H) Leave-one-out analysis plots for TRIM22 on the risk of atrial fibrillation.
